# User-Centered Design Methodologies for the Prototype Development of a Smart Harness and Related System to Provide Haptic Cues to Persons with Parkinson’s Disease

**DOI:** 10.3390/s22218095

**Published:** 2022-10-22

**Authors:** Silvia Imbesi, Mattia Corzani, Giovanna Lopane, Giuseppe Mincolelli, Lorenzo Chiari

**Affiliations:** 1 Department of Architecture, University of Ferrara, 44121 Ferrara, Italy; 2 Department of Electrical, Electronic, and Information Engineering, University of Bologna, 40126 Bologna, Italy; 3 IRCCS Istituto delle Scienze Neurologiche di Bologna, UO Medicina Riabilitativa e Neuroriabilitazione, 40139 Bologna, Italy

**Keywords:** design methodology, inclusive design, human health monitoring, sensory cues, haptic feedback, prototypes, user-centered design, mHealth system

## Abstract

This paper describes the second part of the PASSO (Parkinson smart sensory cues for older users) project, which designs and tests an innovative haptic biofeedback system based on a wireless body sensor network using a smartphone and different smartwatches specifically designed to rehabilitate postural disturbances in persons with Parkinson’s disease. According to the scientific literature on the use of smart devices to transmit sensory cues, vibrotactile feedback (particularly on the trunk) seems promising for improving people’s gait and posture performance; they have been used in different environments and are well accepted by users. In the PASSO project, we designed and developed a wearable device and a related system to transmit vibrations to a person’s body to improve posture and combat impairments like Pisa syndrome and camptocormia. Specifically, this paper describes the methodologies and strategies used to design, develop, and test wearable prototypes and the mHealth system. The results allowed a multidisciplinary comparison among the solutions, which led to prototypes with a high degree of usability, wearability, accessibility, and effectiveness. This mHealth system is now being used in pilot trials with subjects with Parkinson’s disease to verify its feasibility among patients.

## 1. Introduction

Parkinson’s disease (PD) is a neurologic disorder caused by the progressive degeneration of dopamine-producing cells in the substantia nigra pars compacta. The progressive degeneration of these cells results in the four cardinal symptoms of PD: tremor, rigidity, bradykinesia, and postural instability. These symptoms can lead to a large range of physical impairments, including worsened balance and postural control during gait [1].

Postural control involves maintaining, achieving, and restoring a state of balance during movements and with overall posture. All these aspects are impaired in people with PD, affecting their gait [1]. Maintaining balance while walking or standing requires precise control of the head–arms–trunk (HAT) segment of the body [2]. During standing, the postural sway of people with PD shows an increased area, velocity, and jerkiness; their stability limits the maximum center of mass displacements possible without changing one’s base of support, which is reduced, particularly in the backward direction [2]. The characteristic flexed, rigid postural alignment of these patients results in a forward position of the body’s center of mass, possibly to protect against falling backward [3]. In some patients, the maintenance of balance during standing may worsen on levodopa, possibly due to levodopa-induced dyskinesia [4]. Lateral balance control is particularly affected in people with PD; lateral trunk sway is significantly elevated during quiet stance and while walking (with and without obstacles) [5]. While a small amount of lateral movement is necessary for stability and optimized energetics, too much is related to increased falls [6].

Despite pharmacological therapies, postural and gait impairments persist and are related to a reduction in personal independence and safety. Thus, the importance of developing alternative approaches and strategies to manage these problems is clear [1].

Besides drug adjustment, the traditional approach consists of postural rehabilitation with ad hoc exercises [7].

Innovatively, deep brain stimulation and botulinum neurotoxin might have beneficial effects on Pisa syndrome and camptocormia in people with PD [8,9,10].

A different possibility that does not involve surgical treatment and the consequent risk of any side effects is the providing of information on trunk inclination to patients to help improve their postural behavior. Artificial biofeedback systems can provide additional sensory information about trunk inclination to the brain, supplementing natural sensory inputs [10].

### 1.1. Sensory Cues in Rehabilitation of People with PD

The use of external sensory cues (e.g., auditory, visual, or haptic) to reinforce attention toward the task is an effective gait rehabilitation strategy for persons with PD; the cues stimulate the voluntary executive component of action by activating the attentional-executive motor control system and bypassing the dysfunctional, habitual, sensorimotor basal ganglia network [11]. This strategy helps people with PD improve gait consistency and rhythmicity. In the past, auditory cueing during gait has typically been provided continuously in an open loop (regardless of gait performance). These findings have been summarized in several systematic reviews [12].

In the therapeutic context, people are instructed to match foot strike with each beat of the auditory rhythm: these so-called ‘cues’ facilitate the initiation or continuation of the movement. In addition, visual and somatosensory-cueing modalities have been investigated. Visual cues are offered as horizontal lines taped directly onto the floor [13], and somatosensory cues are offered as rhythmical pulsed vibrations applied to a bony structure so that the stepping rhythm can be matched to the vibratory cues [14]. The use of wearable technologies during gait can overcome traditional open-loop cues, providing customized cueing: stimuli are triggered when gait deviates from normality, thus providing patients with immediate feedback on their performance. These closed-loop stimuli (audio, visual, and proprioceptive) are based on the “knowledge of performance”, which is indicated as one of the optimal techniques for motor rehabilitation in PD subjects. In contrast to open-loop systems, in closed-loop systems, the external information does not necessarily become part of the participants’ movement representation, thus possibly decreasing cue-dependency development [15]. The possibility of real-time biofeedback represents an essential step toward the maximum benefit and clinical impact of wearable sensors.

Current experimentations have shown that sensory cues providing temporal and spatial information can facilitate the gait in people with PD by increasing walking speed, stride length, and cadence [12].

Specifically, haptic feedback has shown significant and effective improvements in gait performance, being, at the same time, usable, accessible, user-friendly, and not requiring specific cognitive or physical skills to be interpreted [16].

Haptic feedback systems are generally composed of an actuation system providing vibratory cues to the patient, a sensory system collecting measurements about the user, and a processing unit running an algorithm that suggests when to activate actuators [16,17]. The actuators are usually positioned around the trunk, hips, or head and can number from two to eight, depending on the body position [16,17,18]. The sensors usually integrated into these systems are inertial measurement units (IMU), commonly positioned near ankles, lower trunks, hips, knees, shins, or feet [18,19]. Processing units used in these systems are generally smartphones, computers, and Arduino boards [17,20].

Many real-time biofeedback studies have been performed in laboratories with grounded equipment, demonstrating the clinical benefits of real-time gait retraining. For example, Ginis et al. [21,22] compared the effects of intelligent auditory cueing (IntCue) and intelligent verbal feedback (IntFB) on gait as alternatives to traditional open-loop continuous cueing (ConCue). Those studies showed that, during prolonged indoor walking, both IntCue and IntFB conditions were at least as effective as ConCue for optimizing gait in PD patients [21,22]. Casamassima et al. [23] developed a wearable sensor and smartphone-based system that provided the same IntFB described above to improve the dynamic balance and gait performance of people with PD. Ginis et al. [21] tested the feasibility of this system in a real-life context; they discovered that the wearable system was well-accepted and seemed to be a practical approach to promote gait training in PD subjects.

Even if many studies tried to highlight different rehabilitation roles for biofeedback systems, there are still no certain indications about which kind of biofeedback strategy can implement a specific motor aspect [22].

When considering the wearability factor, not all systems can integrate sensors and actuators in a single compact device [19,24], reducing user-friendliness.

A significant opportunity given by wearable systems is the possibility of collecting data in a more naturalistic environment even if, up until now, most real-time biofeedback studies have been performed in laboratories, demonstrating the clinical benefits of real-time gait retraining [12].

Current studies demonstrate that a crucial aspect needing to be implemented in biofeedback systems is the design of wearable devices, potentially improving device acceptance for subjects with PD. This goal is related to ease of use, accessibility, wearability, and emotional acceptance. Technological innovations need to be empowered by users’ emotional satisfaction to fully express their potential and improve the quality of daily life for people with PD.

These new, real-time systems seem to increase adherence to treatment, self-management, and quality of life, also allowing personalized and tailored rehabilitation for the individual user’s needs [21].

### 1.2. The PASSO Project

This paper shows part of the design process of the PASSO (Parkinson smart sensory cues for older users) project, which aims to design a smart system for the submission of haptic cues to rehabilitate postural disturbances during gait in real-life (including Pisa syndrome and camptocormia). To this extent, a mHealth system based on a wireless body sensor network with a smartphone and different smartwatches (SWs) has been developed. This wearable system enables the real-time extraction of trunk postural features. Thanks to SWs, feedback is returned to the user in the form of haptic cues, encouraging the user to maintain her/his trunk postural behavior or correct it, depending on the clinical needs. The PASSO project follows a user-centered design (UCD) approach, consisting of an iterative three-cycle design process. Each cycle (composed of four phases: planning (P1), analyzing (P2), creating (P3), and verifying (P4)) corresponds to the achievement of a technology readiness level (TRL) [25]. The three cycles address different aspects of the project:

- 1st UCD Cycle (C1): sensory cues and system. TRL 4 (technology validated in a laboratory);
- 2nd UCD Cycle (C2): devices transmitting sensory cues. TRL 5 (technology validated in relevant environment);
- 3rd UCD Cycle (C3): rehabilitation system for PD users. TRL 6 (technology demonstrated in relevant environments) [25].

During the first design cycle, several visual, auditory, and vibratory cues were tested to determine which had more influence on the spatiotemporal parameters related to the gait of people with PD. Auditory cues were sent to users by earphones, visual cues by the lenses of the smart glasses, and vibratory cues by the earpieces of the smart glasses [26].

The second design cycle of the PASSO project is covered in this paper. The goal was to design a mHealth system capable of transmitting vibrations to the person’s body to improve posture, diminishing impairments like Pisa syndrome and camptocormia. Specifically, we describe the development of prototypes for use in this cycle’s testing protocol (P4).

The third and last design cycle will address the development of the system app and interface for the different users involved in the project.

The pervasive application of new digital technologies offers the possibility of contributing to an innovation process that also associates elements of services and communication with products. In the healthcare field, new devices can identify the new typologies of products for fragile users with specific needs. The possibility of developing systems composed of products, services, and processes makes it possible to improve the usability and effectiveness of developed solutions.

In the PASSO project, authors used UCD to properly match qualitative and quantitative aspects of a complex and innovative research field, characterized by the necessity of integrating human sensations and perception to advanced technologies requirements.

UCD is closely related to human–computer interaction HCI, which “focuses on how humans relate to computing products”. UCD is described as “a discipline concerned with the design, evaluation and implementation of interactive computing systems for human use and with the study of major phenomena surrounding them”.

The overall goal of these fields is to improve the user experience, defined in ISO 9241-210:2019 as “a person’s perceptions and responses that result from the use and/or anticipated use of a system, product or service” [27].

## 2. Materials and Methods

For each phase, the authors will demonstrate and justify the design process adopted.

### 2.1. Planning Phase (C2P1)

Results of the previous cycle showed that haptic cues have the potential to improve motor performance; it was thus decided to focus on analyzing how vibratory stimuli can be used for the rehabilitation of posture in the second design cycle. Specifically, a new mHealth system transmitting haptic cues was tested.

#### 2.1.1. Team

This design cycle involved a multidisciplinary mix of professionals:

- Designers (user research, usability, UCD methodology, interaction design, product design, and visual design);
- Medical operators (neurology, physiatry, physiotherapy, geriatrics, etc.);
- Technical operators (biomedical engineering, computer science and technology, informatics, etc.).

#### 2.1.2. Users

Persons identified and engaged in this project were:

- PD USER (US): the typical user with PD is facing the early stages of the disease (Hohen & Yahr I-II), actively combating the degeneration using different cognitive and motor rehabilitation and training techniques under the care of a specialized doctor;
- MEDICAL OPERATOR (MO): the medical operators specialize in movement disorders and PD rehabilitation. Usually, they work on rehabilitating users’ gait and posture to postpone or diminish motor symptoms. They may also conduct trials, experimenting with new devices for their training sessions;
- TECHNICAL OPERATOR (TO): the technical operators comprise a designer, a biomedical engineer, an ICT engineer, and an informatician. They were part of the multidisciplinary team developing the PASSO project.

#### 2.1.3. Context

The scientific literature indicates that the use of smart devices for the transmission of sensory cues, particularly by means of vibrations on the trunk, is a promising feedback technique to improve peoples’ gait performance. The devices have been used in different environments and are well-accepted by users [26]. In addition, vibratory feedback does not require fine physical senses that may deteriorate with age, such as vision or hearing. For these reasons, it is desirable to experiment with vibratory cues to improve balance, avoid falls, or mitigate freezing of gait in Parkinson’s subjects (FOG). The sensors’ location is a crucial aspect of this kind of signal. The inertial sensors (IMUs) are commonly positioned in various locations, while haptic feedback actuators are usually separately located (in a different body area). The most common placement area for sensors providing vibrotactile feedback, specifically for balance, is the trunk at waist level [21].

Prototypes were created to test the system in an ambulatory context under the supervision of a medical operator. The intent was to further develop the system so that it can be used by persons with PD in their everyday environments (indoor and outdoor) as they perform their daily activities.

### 2.2. Analyzing Phase (C2P2)

#### 2.2.1. Users’ Requirements

Preliminary, informal interviews with the USs, MOs, and TOs were conducted to determine users’ needs in terms of their expectations and fears regarding the system. Specifically, participants involved in this specific phase of the research project were 2 PD users presenting different significant symptoms of the disease, 2 MOs with expertise in rehabilitating therapies for gait and postural disturbances, and 10 TOs specializing in design for healthcare purposes or biomedical engineering. It is important to specify that the experimentation involved fewer PD subjects than planned due to pandemic restrictions making it difficult to involve patients in nonclinical trials. Each participant, after testing the prototypes, was singularly interviewed and asked to express his/her requirements and expectations about the project development.

The following table lists the needs described during the interviews (Table 1). For each need, a code was created, and the type of user who described it was annotated. The needs are grouped according to the category they refer to. The list of domains was elaborated, starting from a list describing the common needs of older users regarding the use of smart garments [28].

#### 2.2.2. Technology Selection

The developed system is supposed to be used primarily in the ambulatory environment. Medical operators need a system that helps them train patients’ gait and posture; therefore, they were involved in the project as secondary users. The wearable device uses haptic cues as the rehabilitation treatment to correct the frontal or mediolateral inclination of the person’s trunk.

The system could also be used in the user’s daily life; after a posture rehabilitation phase supervised by a medical operator, a simplified version of the system could be left with the user for training during normal daily activities in a realistic context.

The system consists of two parts: a sensor to detect maladaptive movement and an actuator to provide vibratory feedback. The sensors’ location is a crucial part of the design process since it has a considerable impact on the wearability of the system. According to Gonçalves et al. scientific literature review (2021), wearable sensors are most commonly located at the ankle, waist, shin, or foot insole, while the vibratory actuators are usually separately located—at the head, trunk, waist, hip, wrist, ankle, or foot insole [26]. The location of the actuator is also very important; they should be near bony structures so that the signal is clearly perceptible—but they should avoid being annoying, uncomfortable, or stressful. The ideal locations for both parts can optimize the system’s usability. Different locations were evaluated to determine which could be perceived better.

A commercial Android SW was chosen to function as both a sensor and actuator, creating a system that is effective and wearable. The SW was fastened to a wearable cloth to ensure the correct positioning of the unit. It is important to note that the SW must be positioned precisely in order to correctly identify the trunk inclination of the subject and provide a perceptible haptic cue.

During this phase, the peculiarities of primary and secondary users (PD persons and medical and technical operators) were analyzed to understand their most important needs regarding the smart device and the system’s design. Through private interviews and group meetings, the human and technical requirements of the project were identified and discussed.

#### 2.2.3. Quality Function Deployment

**Figure 1:**
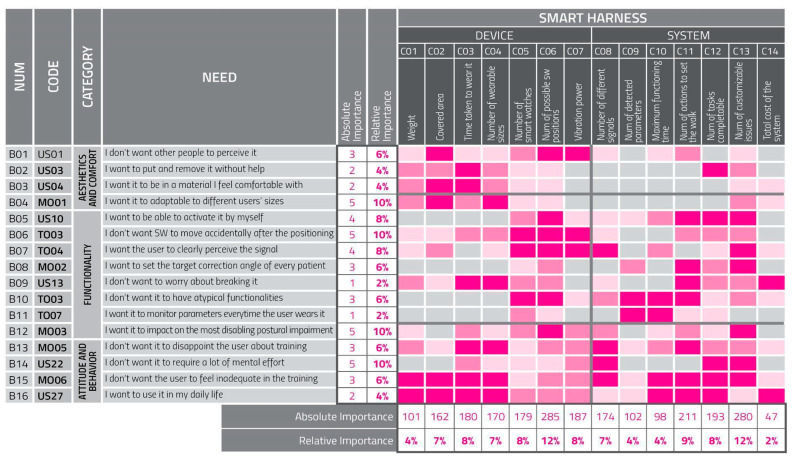
Quality function deployment matrix for the smart harness. Relationships between needs and characteristics are defined to evaluate their impact on the whole project.

It was possible to select some of the needs expressed by the user groups and evaluate their relationships to some measurable characteristics of the smart device and the system developed in this design cycle. The characteristics were selected with the help of the TOs to ensure their relevance. The quality function deployment (QFD) tool, primarily conceived for the evaluation of design strategies in companies, was modified to be more accessible to the multidisciplinary team members [29]. It was used to create a hierarchy matrix of the project’s characteristics in light of users’ needs, highlighting the characteristics with the highest potential for innovative and pervasive impacts on the project [26]. The whole team was then asked to score each need so that the ones to be inserted in the matrix would be the most impactful. After this step, the team scored the degree of relationship between every need and every characteristic (Figure 1). The scores were reported at the intersections of lines (needs) and columns (characteristics) using the following classification.

- 0 (light gray): the cue does not respond to the need;
- 1 (light pink): the cue weakly responds to the need;
- 3 (pink): the cue satisfactorily responds to the need;
- 9 (magenta): the cue strongly responds to the need.

The use of colors helped team members use and interpret the QFD matrix (even if they were not experts). The QFD algorithm provided percentages representing each characteristic’s relative and absolute importance with respect to the others in the matrix [29].

The QFD scores were an important reference for the development of the design and technical solutions [29]. Table 2 ranks the characteristics by their relative and absolute importance.

An examination of the QFD scores reveals that characteristics relating to customizable physical features and system settings have the most impact on the project’s rated usability. On the other hand, the device’s weight and energy autonomy and the cost of the whole system were deemed the least impactful.

It is interesting to note that, considering that only the QFD scores were given by users with PD, those needs that are considered more important were the ones related to functionality (often expressed from other categories of users).

## 3. Results

### 3.1. Creating Phase (C2P3)

Different positions on the back, shoulders, and chest were tested to determine the optimal location to feel the vibration, considering its positive impact on trunk postural control.

The haptic cues were sent on demand via an ad hoc multi-platform app, using the SW as an actuator (to mimic the operation of the final mHealth system); the smartphone sent the sensory cues on demand.

**Figure 2:**
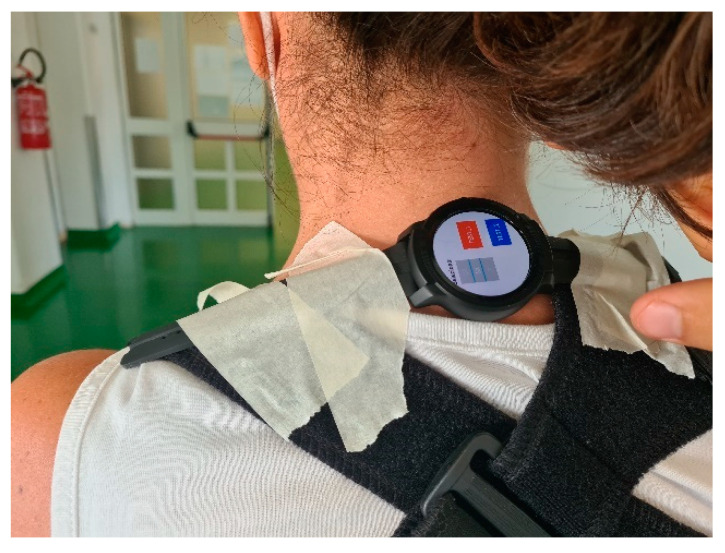
One option for smartwatch positioning.

The primary experimenter was the medical operator who would be setting the training protocols for people with PD (Figure 2).

**Figure 3:**
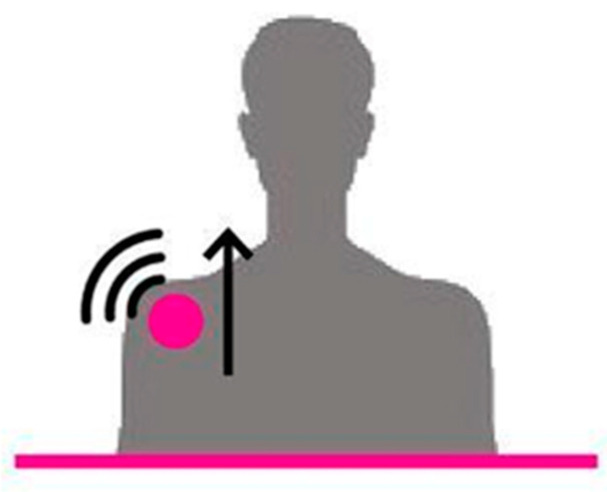
When the cue was submitted to the correct mediolateral inclination, the affected (lowered) shoulder was raised.

According to the medical operator, the intention was to make the person straighten their trunk when leaning too far forward or when it was unbalanced on the impaired side (Figure 3).

We developed a harness with a modified band across the shoulders and back, which guaranteed that the SWs were positioned so as to correctly detect the trunk inclination and provide an effective haptic cue.

The appropriate cue, consisting of a single short vibration, was determined after the first trial with the medical operator.

Different actuator locations were tested to determine which would be the most perceptible and least bothersome for the user. The wireless system made these tests trivial since the SWs only needed to be moved to the desired locations.

**Figure 4:**
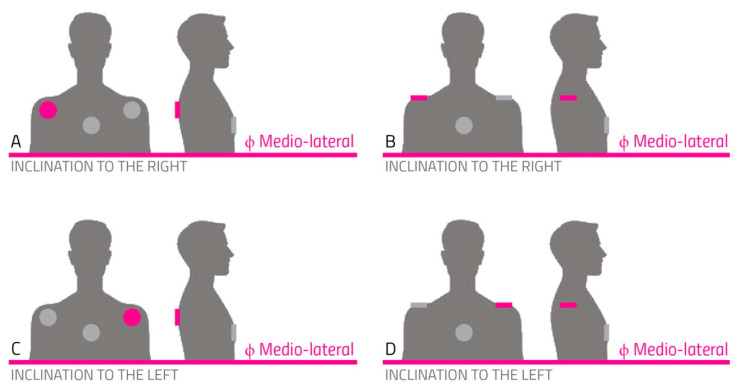
Actuator positioning for the correction of mediolateral inclination.

To rehabilitate mediolateral trunk inclination (as represented in Figure 4), the SWs were put against the shoulder blade or the shoulder. These positions were directly on the bones, guaranteeing a better cue transmission.

Both solutions were reasonably effective, so it was decided to leave the choice up to the users, depending on their personal preferences.

**Figure 5:**
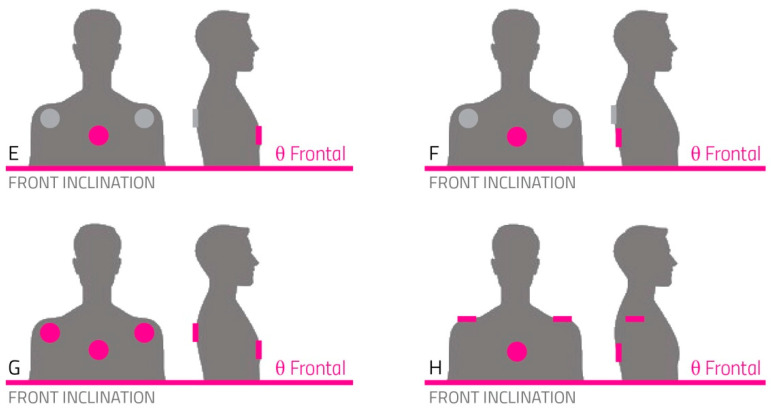
Actuator/SW positioning for the correction of frontal inclination.

On the other hand, to restore correct frontal inclination, we tested configurations involving one single actuator centrally positioned on the sternum or the back, which was also proximal to the bone, as represented in schemes E and F in Figure 5. The other solution, involving three SWs (schemes G and H), was dismissed because it was perceived as too intense and annoying to correct frontal trunk inclination.

The solutions were used as inputs for several prototypes in order to determine the best locations that made the user feel comfortable.

Initially, the intended design was a type of shirt incorporating SWs as actuators, transmitting the vibratory cue. However, it became clear that the solution needed to be improved; it was difficult to position the SW faces correctly on different body shapes, and the low pressure exerted by the fabric on the devices interfered with any contact with the body and the perception of the signal.

Therefore, it was decided to develop a tailored harness inspired by an existing postural band that pulls the shoulders back. This solution guarantees the more precise positioning of the SWs and better contact with the body, allowing improved perception of the signal while helping with postural training due to its function.

Three types of sensory cues were initially developed:

- Single short vibration;
- Single persistent vibration;
- Intermittent vibration.

After the first application was overseen by the MO, it was decided to use the single short and intermittent vibrations to correct postural inclination. The single persistent vibration was considered anesthetizing, so it was dismissed. The vibration was applied to the shoulder that was supposed to lift up, or on the chest or back for forwarding inclination.

### 3.2. Harness Prototypes

Several prototypes were developed.

Various position configurations on the back, shoulders, and chest were tested using the help of the neurologist who would be setting the training protocols for the US.

**Figure 6:**
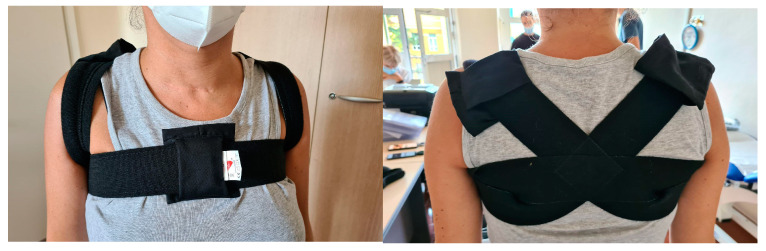
First smart harness solution positioning SWs on shoulders and chest.

Two solutions were selected:

The harness (Figure 6) can hold up to three SWs: two lateral ones at the shoulder blade or on top of the shoulder and the central one on the sternum. This configuration can transmit a single vibration to the chest or three simultaneous vibrations to the chest and shoulders. This solution was intended for users who tend to lose their balance in the frontal plane only.

**Figure 7:**
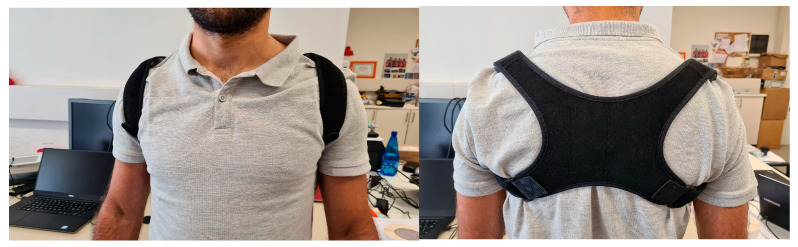
Second smart harness solution positioning SWs on shoulders and back.

The second solution (Figure 7) differs from the first because it locates the central SW near the first thoracic vertebra instead of on the chest. This design encourages better posture, making the most of the effect offered by the postural band. This model, which is primarily intended for people who tend to lose their balance along the lateral plane, allows different positioning options for SWs in the shoulder area.

### 3.3. System Prototype

**Figure 8:**
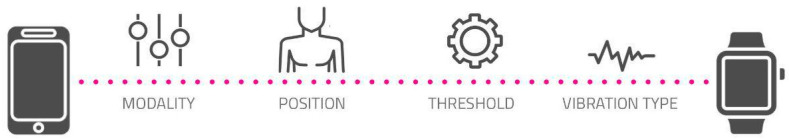
System scheme showing data transmitted from the smartphone to the SW.

Figure 8 illustrates the scheme of the mHealth system. A smartphone acts as a remote control to set the different use modalities and start/stop the SW, which is the system’s core. The SW acts as a sensor and actuator, returning the haptic-trunk biofeedback in real time.

After the SW is started using the smartphone, the system guides the user with voice instructions. Initially, it reminds the subject to maintain the target posture; once this calibration phase is completed, the system will tell the user that he/she can start walking. Based on this calibration phase and the threshold set by the clinician, the SW will trigger the corrective haptic feedback to help the subject return to his/her target posture.

The planned corrections deal with two trunk postural impairments: mediolateral and frontal-trunk inclination. For the former, it was decided to make the SW vibrate as a signal to raise the lower shoulder. When correcting for frontal sway, vibrations on the chest or back alone were applied for a mild effect; for a more substantial stimulation, multiple vibrations involving all three actuators at the same time were applied.

The inertial data files from the three-axial accelerometer and the three-axial gyroscope, along with the file with the trunk angle information, are stored in the SW memory to allow offline data analysis.

The SW app creates a new folder for each day’s test; the SW folder contains the three.txt files with sequential numbers generated in each daily test. These files can be imported into different software for data analysis (Excel, MATLAB, etc.). The file with the “_Rot” extension has the trunk angle information. Its first two rows have settings information, such as the position (AP = “antero/posterior”), relative threshold in degrees, and absolute time. Starting with the third row, three columns display the pitch (θ) and roll (ϕ) trunk angles in degrees and the sample time difference in nanoseconds, respectively.

#### Algorithm for Estimating Real-Time Trunk Inclination

Depending on the application, different solutions are used to estimate the trunk angle from the inertial and magnetic sensors (M-IMU). Data provided by body-fixed M-IMU sensors are affected by noise- and time-varying biases; therefore, the use of algorithms is necessary to process the data and obtain a smooth, bias-free estimation of the trunk inclination [30]. Our mHealth system uses a sensor fusion approach, which obtains the anterior and medial-lateral trunk inclination (pitch (θ) and roll (ϕ) angles, respectively) from the accelerometer and gyroscope.

First, the raw inertial data were low-pass-filtered to reduce noise (corner frequency from 2 to 5 Hz) before the angle calculations.

Pitch (θ) and roll (ϕ) angles were then calculated from the accelerometer output following a trigonometry approach. These angle estimations, ideal for real-time applications, can be calculated quickly, but they cannot be applied in dynamic activities like running or walking; their applicability is limited by the singularity of the trigonometric function, which leads to the gimbal lock issue [30]. For this reason, we also considered the raw data output by the gyroscope, which can be integrated to produce angle estimates. However, the integration accumulates noise and offsets over time, which drift. The problem of drift is commonly addressed by starting the task in a stationary position for a few seconds to reduce angular velocity noise and then subtracting this noisy term from the subsequent measurements. A second option is to use accelerometers to compensate for the linear drift in the gyroscope integral when the inertial sensor is quite still. A combination of these two techniques has been used for the real-time estimation of pitch and roll angles during various motor activities in different contexts [31].

Our mHealth system uses this combination to provide reasonable, real-time pitch (θ) and roll (ϕ) angles.

It is important to note that each Android device has different built-in IMUs with different sample frequencies. The frequency changes around a fixed value depending on the Android operating system. This value is related to the CPU capabilities of the specific device. In our mHealth system, we adopted the Mobvoi TicWatch E2 [32] as our SW. We set a dynamic sample frequency of about 50 Hz, which is sufficient enough to ensure correct operation without overloading the system CPU—thus avoiding delays in biofeedback stimulation.

## 4. Validation Process

### 4.1. Verifying Phase (C2P4)

#### 4.1.1. Testing Prototypes with Users

This design phase directly involved PD users. Their participation was necessary to ensure that the device could provide useful feedback in the presence of real posture impairments.

**Figure 9:**
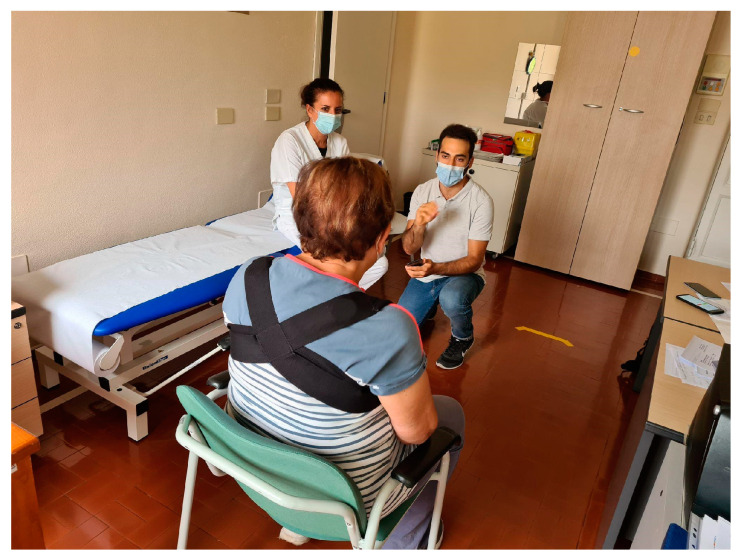
Picture of a patient during the ambulatory trial testing the smart harness’s wearability.

Users were studied in ambulatory and laboratory contexts while wearing and using the smart devices. They were asked to comment on the ergonomics and wearability of the smart harness (Figure 9). They were also asked to give feedback about their perception of the vibrations, both from physical and cognitive points of view.

After testing different configurations, we observed that cues from a single SW at a time were the clearest and most easily interpreted. This observation supports an important clinical concept: for a patient with PD, it is important to define one and only one clinical target to improve the most impaired function. Attempts to correct more than one gait or posture aspect at the same time could cause cognitive overload, making the PD person feel overwhelmed. On the other hand, focusing on a single task improves the user’s performance [33].

For example, for subjects with Pisa syndrome, it is enough to have one SW on the compromised side to act as a reminder every time it is needed. The developed system allows a great deal of customization, so it can target specific clinical needs.

Testing the prototypes with the users confirmed that the ability to customize several functions must be included in the project. The two models were perceived differently by different users, depending on their body shape. It became clear that the smart harnesses needed to be adaptable to different body shapes and sizes; further, some women did not feel comfortable wearing the model with the chest band. Another practical aspect is that the model must accommodate different locations of SWs (on top of the shoulder or on the shoulder blade), considering the user’s skin sensitivity and fat mass.

#### 4.1.2. Pitch and Roll Accuracy within the Usage Scenario

When verifying the reliability of our trunk inclination algorithm, two standing balance tests were performed to compare the SW Mobvoi TicWatch E2 and the smart harness together against a gold standard.

The gold standard is the built-in rotation vector sensor available in recent smartphones. We used the Samsung orientation sensor in the Samsung Galaxy M31s. The sensor uses a sensor fusion approach that merges the information from the IMU and magnetometer to improve accuracy [34].

This approach reports orientation using quaternion coordinates, which is characterized by a small number of numerical integration errors and does not require the computation of trigonometric functions. Notably, the quaternion representation avoids the gimbal lock singularity. A deeper analysis and explanation of quaternions can be found in work by Sabatini (2011) [31]. Unfortunately, due to its computational cost, the real-time built-in Android rotation vector sensor is currently only available in mid- to high-quality smartphones, not in SWs. However, given probable technological advances in the future, this method could also be handled by SWs.

**Figure 10:**
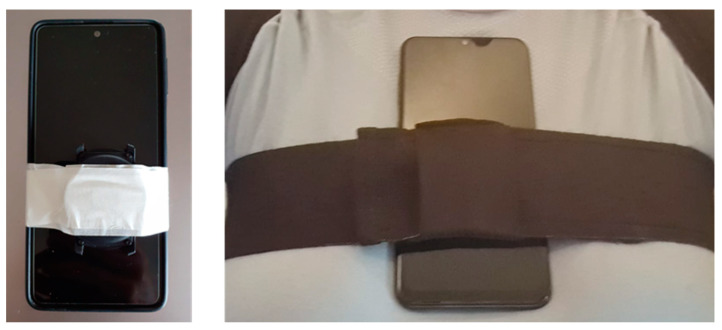
Setup of the reliability test during forward inclination. The SW Mobvoi TicWatch E2 and the smart harness developed together were compared to the gold standard system (smartphone with MATLAB mobile platform).

In our agreement evaluation, the forward and the mediolateral trunk inclination were tested. In the first test, we assessed three different forward inclinations (of about 15°, 20°, and 30° degrees) from a standing position, while in the second one, six different mediolateral inclinations (of about ± 10°, ± 15°, ± 20°) were assessed. As noted, the trunk angle information is stored to allow offline data analysis, but in this case, we used the MATLAB mobile platform [35] to get the gold standard orientation from the Samsung Galaxy M31s. We set it to the same dynamic sample frequency as the SW (50 Hz). Figure 10 illustrates the setup of the reliability test during forward leaning.

Data analysis was performed using MATLAB (version 2018a, Mathworks Inc., Natick, MA, USA). As a preliminary step, we resampled the data of both devices, using the resample function [36], with a fixed sample rate of 25 Hz. We then manually synchronized the two devices. We removed the initial offset for both systems.

**Figure 11:**
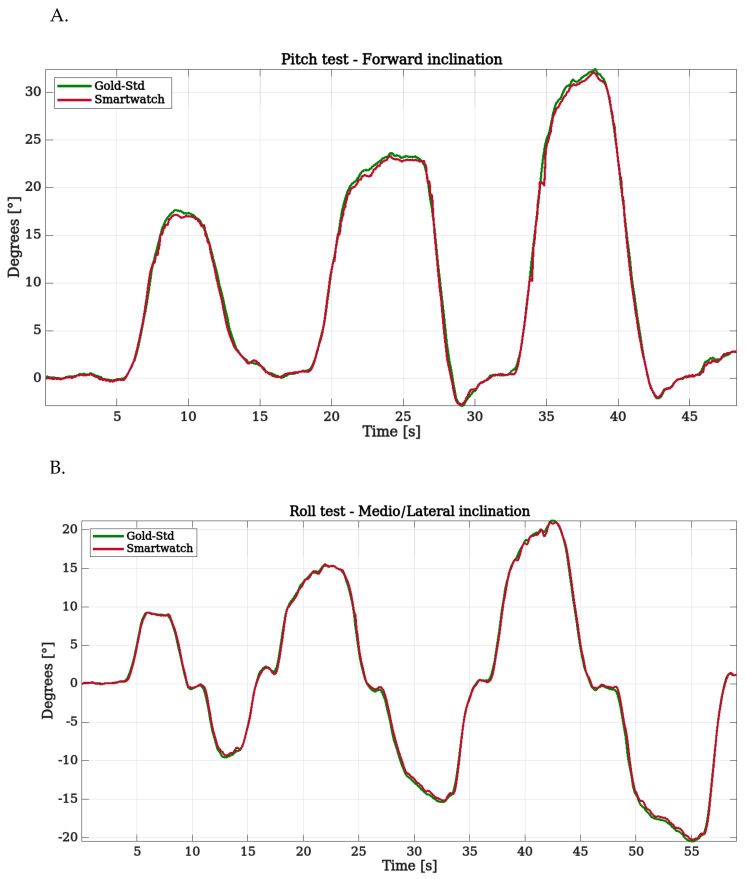
(**A**). Qualitative sensor validation for the pitch angle (θ). (**B**). Qualitative sensor validation for the roll angle (ϕ).

An initial qualitative sensor validation check was performed by graphically comparing the two estimations. The differences between the two estimations for both pitch and roll were minimal, as illustrated in Figure 11.

**Figure 12:**
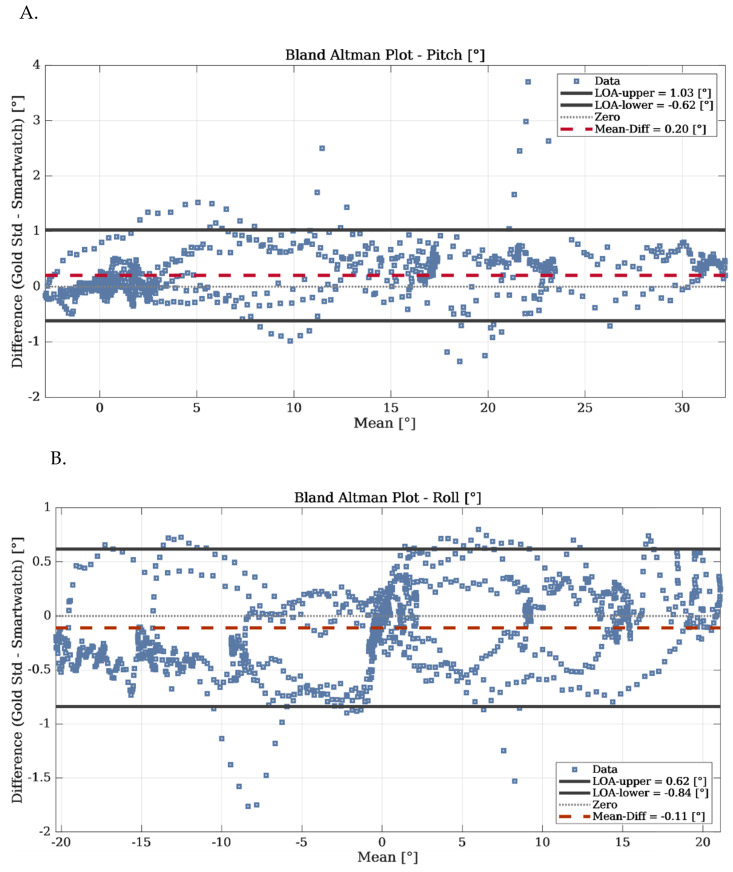
Bland–Altman plots illustrating differences for (**A**). the pitch and (**B**). the roll angles, estimated with our SW real-time algorithm and the built-in rotation vector sensor in the Android Samsung Galaxy M31s.

We used Bland–Altman plots to compare the estimations (see Figure 12). The differences between the pitch and roll angles obtained with our SW algorithm and with the gold standard are reported.

The results show good agreement between the two methods; the bias is close to zero and the SD is within 1° for both pitch and roll angles.

## 5. Discussion

In this work, we presented the second design cycle of the PASSO project. The specific aim is to design a smart system for the transmission of haptic cues to rehabilitate PD impairments such as Pisa syndrome and camptocormia. The design process of a new mHealth system based on a wireless body sensor network with a smartphone and different SWs has been developed to achieve this aim. Thanks to SWs, this portable wearable system enables the real-time extraction of trunk postural features. The users receive biofeedback in the form of haptic cues, which encourage them to maintain/correct their trunk postural behavior as needed. Compared to other recent wearable solutions proposed for gait rehabilitation of PD subjects, our mHealth system uses SWs as an innovative aspect; this allows for the combination of a single physical device for the haptic actuator and the processing unit, giving the user the advantage of wearing a single compact smart object. The smartphone, in this case, is a graphical interface of the mHealth system, facilitating the user in starting and stopping the training session and visualizing a data report about it.

The choice of wearing the haptic actuator and the sensor unit in the shoulder area to treat camptocormia and Pisa syndrome in PD subjects was unexploited in recent works [19,20,21,22,23,24].

Interestingly, during the testing phase, it was observed that multiple stimulations could confuse the user making him/her feel uncomfortable and overwhelmed. That is why, after several proofs, it was finally decided to use only one SW at a time, correcting only one typology of inclination at a time. When users incline both in the frontal and mediolateral plans, it is preferable to intervene only on the inclination evaluated as higher. Training to correct this single aspect will consequently lead to a decrease in the inclination in the other plane.

The system architecture allows easy customization according to the different clinical needs of different users. Reassuringly, the verifying phase showed that our SW real-time algorithm is reliable and accurate enough to estimate forward and mediolateral trunk inclination. Nevertheless, this mHealth system does not require the highest accuracy. Since its intended use is as a postural rehabilitation system, it does not need to be as accurate as an assessment system.

Personalization is fundamental in smart devices addressed to PD subjects. The used smart harnesses were adjustable in size, increasing the fit and wearability of the wearable device. However, they were also perceived differently by users, depending on their bodies (e.g., women preferred the second smart harness because it did not have the part pressing the chest). Even the positioning of the SW in the smart harness was not felt in the same way by different users, e.g., in the case of frontal inclination, most of the subjects preferred to have the SW positioned on the chest, but some others felt uncomfortable with this configuration and preferred the one with the SW on the back between shoulder blades.

Moreover, an important issue is the possibility of the system being used in the user’s daily context for rehabilitating sessions on his/her own (reports of which will, however, be delivered to the medical operator), giving the person a sense of familiarity with the device, improving its correct use and encouraging its adoption over the long-term perspective.

Compared with other solutions addressed to PD persons [19,20,21,22,23,24], a potential advantage of this mHealth system is the continuous training available for an extended period in a real-life scenario, which is a key element in the rehabilitation of chronic diseases such as PD.

However, the results obtained here with the limited number of tests and subjects involved in this study need to be confirmed for a larger cohort, adopting a clinical trial protocol in the future.

### Future System Developments

This mHealth system is now being used for pilot trials with subjects with PD. The results will be employed to verify the feasibility among patients and adjust the system for more extensive clinical usage.

Further, the postural trunk features measured by the system may also be exploited in the future to assess postural control, both in a clinical context and—more interestingly—in an environment outside of the laboratory (e.g., at the patient’s home).

Thanks to the logging capability of the system, there is the possibility of evaluating the overall postural performance immediately after the training session. It is also possible to monitor the state of the user automatically over several days. In fact, users’ ability to respond to the haptic feedback and adjust their postural behavior is stored within the device; the information can be evaluated after the treatment or even after multiple sessions.

Adopting a different and innovative approach, a recent study proposed a federated learning-based health monitoring system that identifies and observes patients’ activities and movements using wearable systems. Thanks to this approach, unlabeled data received from the wearable devices are effectively labeled by a deep reinforcement learning algorithm. This approach showed a promising reduction in computation costs, memory usage, and transmission data in comparison to traditional methods [37].

Before reaching the market, each mHealth solution must comply with the new regulations on medical devices in Europe (MDR [EU] 2017/745) in order to improve patient safety. On the other hand, these regulations might limit the development and release of new solutions and software [38].

In addition, the privacy and security of the sensitive medical information of the user must be guaranteed. To this end, more efforts are needed to develop algorithms to ensure highly secured communication. In the future, the use of blockchain technology may guarantee this aspect [37].

## 6. Conclusions

UCD has proven to be an effective tool for managing and organizing the design process. The experiments carried out with the help of primary and secondary users led to satisfactory qualitative solutions, ensuring that the system has a high degree of wearability, usability, and accessibility.

The whole multidisciplinary team was involved in the elaboration of the QFD matrix; thanks to graphic strategies, it was possible for the members to understand the rankings of the members in different research fields. The QFD tool outputs a ranked importance list of measurable characteristics of the smart device and system. The results to date confirm the hierarchy expressed by the QFD.

Prototypes of the system were tested by users in an informal, qualitative way to obtain preliminary results related to ergonomics and usability. These validated results will be used in the final testing protocol to collect quantitative information.

This mHealth system is now being used for pilot trials with subjects with PD. The results will be employed to verify its feasibility among patients and adjust the system for more extensive clinical usage.

A potential advantage of this solution is the continuous training that is available for an extended period in a real-life scenario, which is a key element in the rehabilitation of chronic diseases such as PD.

Further, the postural trunk features measured by the system may also be exploited in the future to assess postural control, both in a clinical context and—more interestingly—in environments outside of the laboratory (e.g., at the patient’s home).

## Figures and Tables

**Table 1:** Users’ needs listed by category.

Category	Code	Need	User Expressing THE Need
Aesthetics Anxiety	US01	I don’t want other people to perceive it	PD user
	US02	I don’t want it to seem like a medical device	PD user
	US03	I want to put it on and remove it without help	PD user
Comfort	US04	I want it to be in a material I feel comfortable with	PD user
	US05	I don’t want it to reduce my mobility	PD user
	US06	I want it to be my size	PD user
	TO01	I want to be able to change size for every user I test	Technical operator
	MO01	I want it to be adaptable to different users’ sizes	Medical operator
Functionality Issues	US09	I want it to last as long as I need it	PD user
	US10	I want to be able to activate it by myself	PD user
	US11	I don’t want the equipment to suddenly stop functioning	PD user
	TO02	I don’t want to risk the watches falling out.	Technical operator
	TO03	I don’t want it to have atypical or unexpected functionalities	
	TO04	I don’t want smartwatches to move accidentally after being positioned	Technical operator
	TO05	I want the user to clearly perceive the signal	Technical operator
	MO02	I want to set the target correction angle of every patient	Medical operator
	MO03	I want it to impact the most disabling postural impairment	
Technology Anxiety	US12	I don’t want to be apprehensive about using it	PD user
	US13	I don’t want to worry about breaking it	PD user
	US14	I don’t want it to interfere with other aids or devices (e.g., pacemaker, hearing aids, etc.)	PD user
	TO06	I want it to be compatible and integrable with Android systems	Technical operator
Perceived Ubiquity	US15	I want it to provide me with communication and connectivity, anytime and anywhere	PD user
	TO07	I want it to monitor parameters every time the user is wearing it	Technical operator
	MO04	I want to use it for healthcare and wellbeing purposes	Medical operator
Resistance	US16	I don’t want it to change the way I interact with other people	PD user
	US17	I don’t want it to change the way I currently live	PD user
	MO05	I don’t want it to disappoint the user when training	Medical operator
Perceived Usefulness	US18	I want it to improve my quality of life	PD user
	US19	I want it to make my life more convenient	PD user
	US20	I want it to make me more effective in my life	PD user
Perceived Ease of Use	US21	I want it to be easy to use	PD user
	US22	I don’t want it to require a lot of mental effort	PD user
	MO06	I don’t want to make her/him feel inadequate in the training	Medical operator
Attitude	US23	I want to think that it is beneficial to me	PD user
	US24	I want to have a positive perception of using it	PD user
	US25	I want it to make me feel safe	PD user
	MO07	I don’t want to frighten her/him proponing the system testing protocol	Medical operator
Behavioral Intention	US26	I want to use it in the future	PD user
	US27	I want to use it in my daily life	PD user
	US28	I want my parents to appreciate my improvements	PD user

**Table 2:** Final list of characteristics’ relative and absolute importance.

Code.	Characteristic	Element	Relative Importance	Absolute Importance
C06	Number of possible SW positions	Device	285	12%
C13	Number of customizable settings	System	280	12%
C11	Number of actions to set the walk	System	211	9%
C12	Number of tasks completable autonomously	System	193	8%
C07	Vibration power	Device	187	8%
C03	Time taken to wear it	Device	180	8%
C05	Number of smartwatches	Device	179	8%
C08	Number of different signals	System	174	7%
C04	Number of wearable sizes	Device	170	7%
C02	Covered area	Device	162	7%
C09	Number of detected parameters	Device	102	4%
C01	Weight	Device	101	4%
C10	Maximum functioning time	System	98	4%
C14	Total cost of the system	System	47	2%

## Data Availability

Not applicable.
